# Supplementary material for: Evaluation of Nationwide Oral Mucosal Screening Program for Oral Cancer Mortality among Men in Taiwan
Source: Int J Environ Res Public Health. 2022 Nov 2;19(21):14329. doi: 10.3390/ijerph192114329 (PMC9654467; doi:10.3390/ijerph192114329)
Supplement: Supplementary file 1 [file ijerph-19-14329-s001.zip › ijerph-1940016-supplementary.pdf]

Supplementary materials

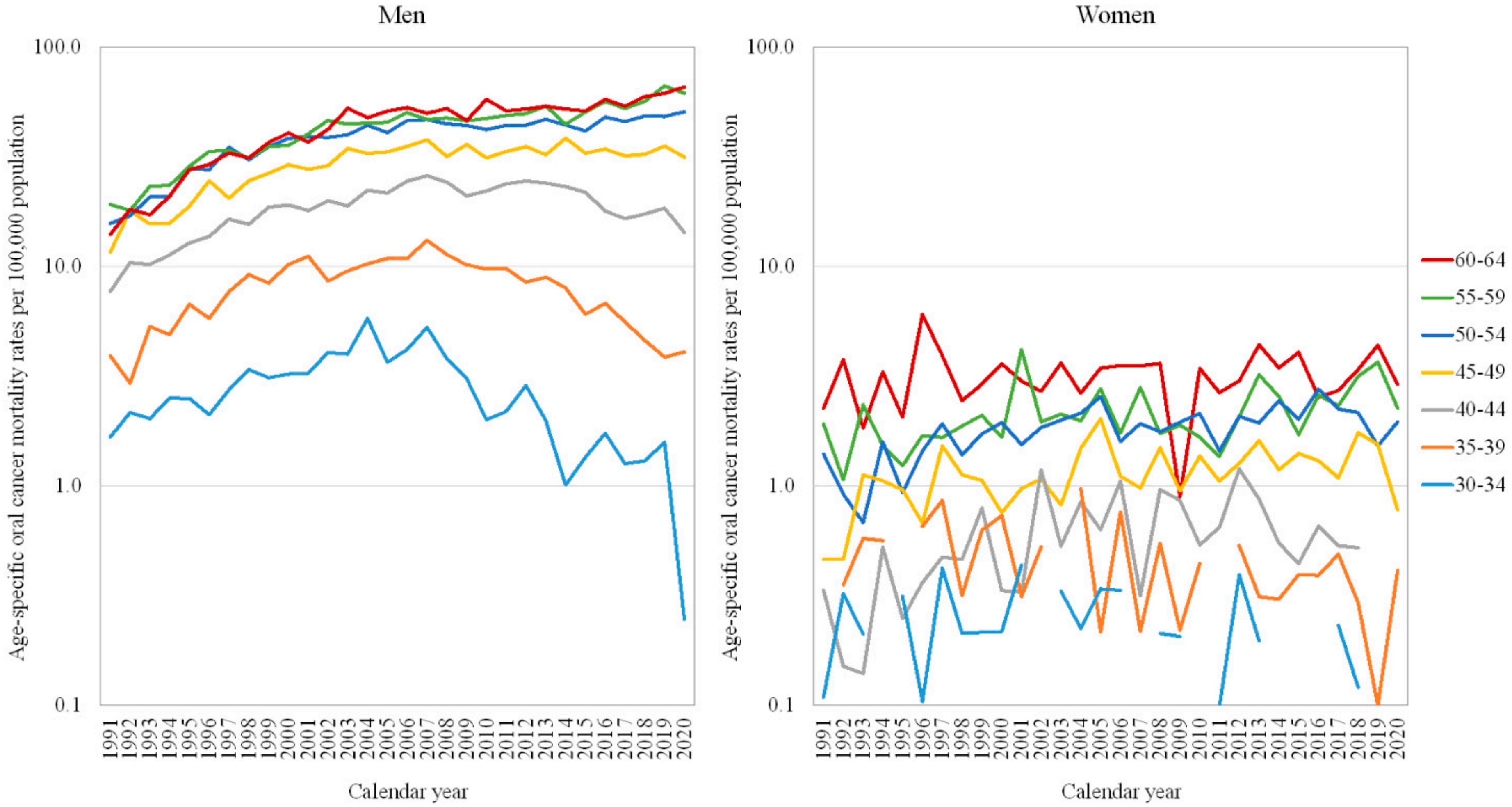

Figure S1. Age-specific oral cancer mortality rates from 1991 to 2020 in Taiwan for men and women.

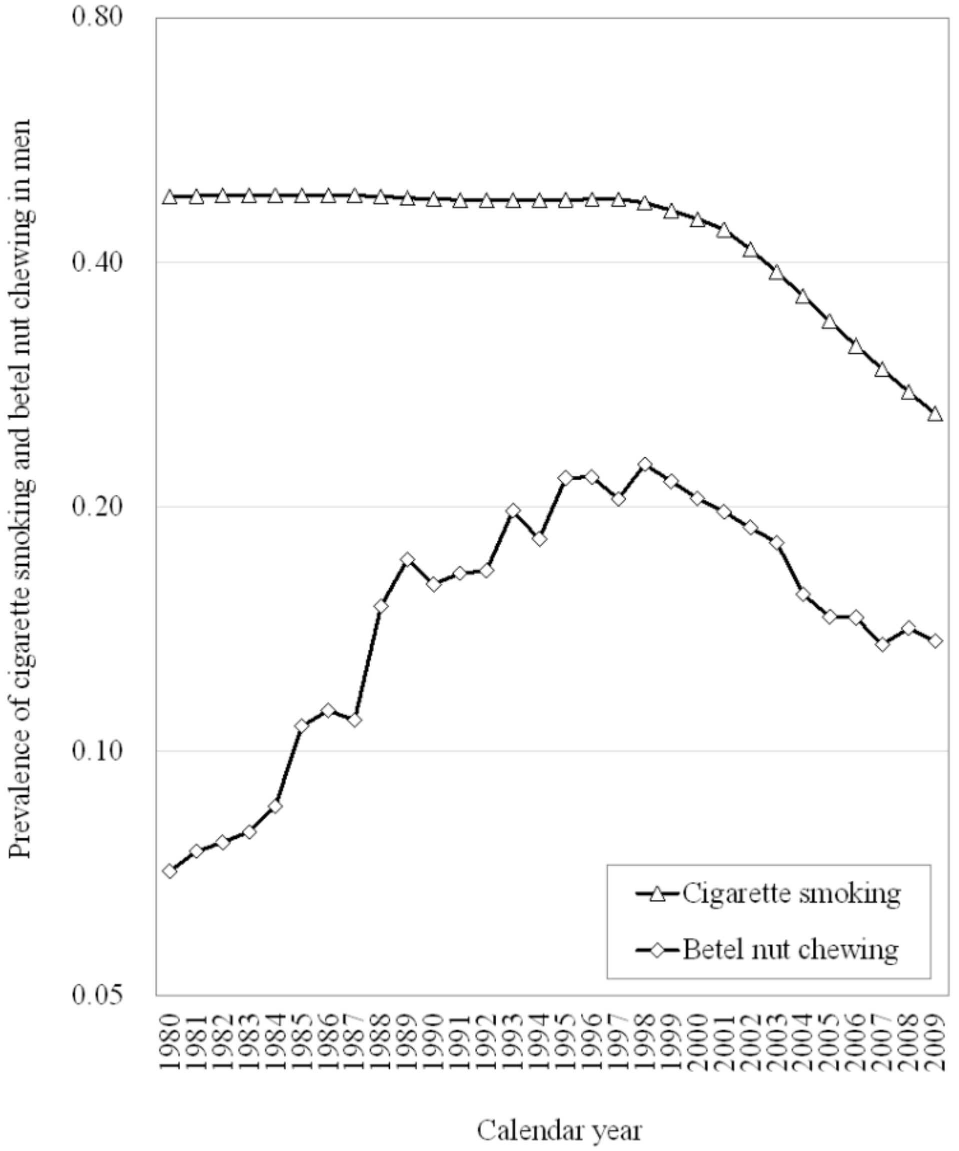

Figure S2. Prevalence of cigarette smoking and betel nut chewing from 1980 to 2009 among men in Taiwan.

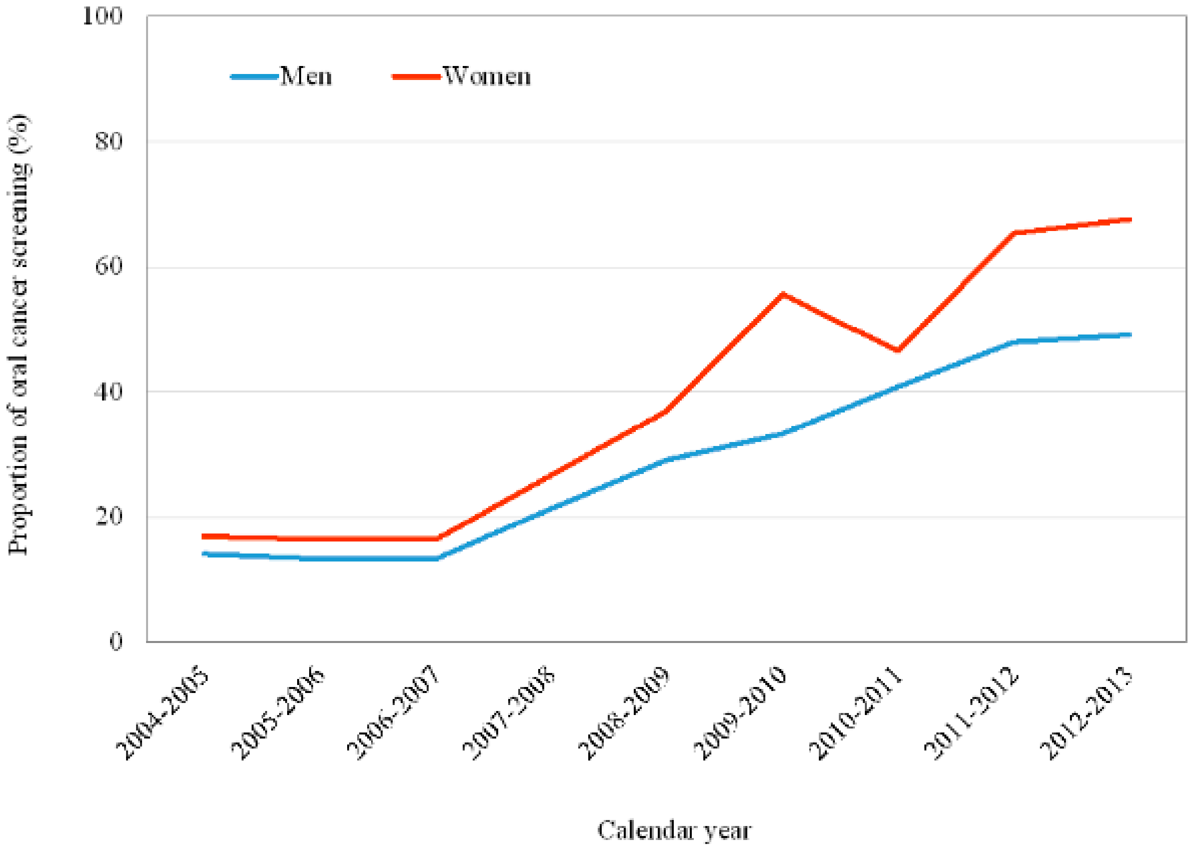

Figure S3. Nationwide oral mucosal screening coverage rates for men (blue line) and women (red line) in Taiwan

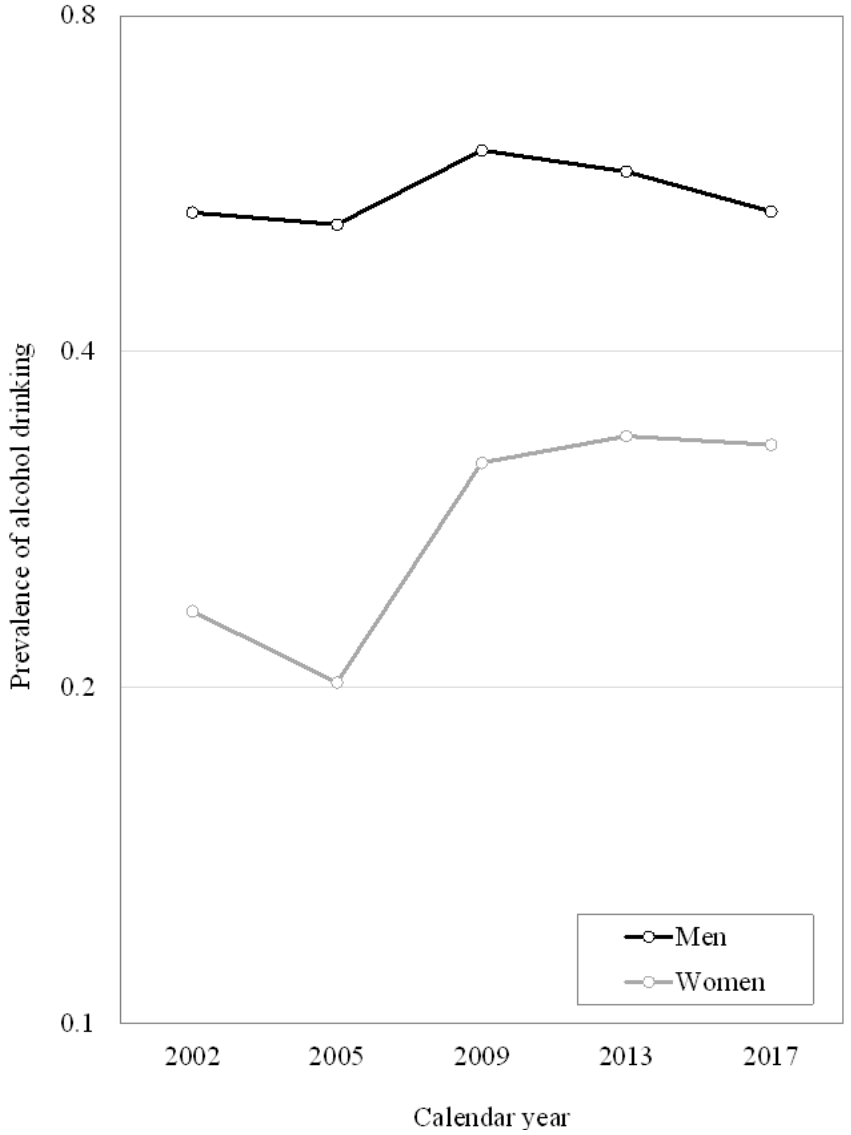

Figure S4. Prevalence of alcohol use from 2002 to 2017 for men and women in Taiwan.
